# The effects of neoadjuvant chemotherapy and interval debulking surgery on body composition in patients with ovarian cancer

**Published:** 2020-11-11

**Authors:** John Vitarello, Marcus D. Goncalves, Qin C. Zhou, Alexia Iasonos, Darragh F. Halpenny, Andrew Plodkowski, Emily Schwitzer, Jennifer J. Mueller, Oliver Zivanovic, Lee W. Jones, Karen A. Cadoo, Jason A. Konner

**Affiliations:** 1 Department of Medicine, Beth Israel Deaconess Medical Center, Boston, MA, USA,; 2 Division of Endocrinology, Department of Medicine, Weill Cornell Medicine, New York, NY, USA,; 3 Department of Epidemiology and Biostatistics, Memorial Sloan Kettering Cancer Center, New York, NY, USA,; 4 Department of Radiology, Memorial Sloan Kettering Cancer Center, New York, NY, USA,; 5 Cardiology Service, Department of Medicine, Memorial Sloan Kettering Cancer Center, New York, NY, USA,; 6 Gynecology Service, Department of Surgery, Memorial Sloan Kettering Cancer Center, New York, NY, USA,; 7 Department of Obstetrics and Gynecology, Weill Cornell Medical Center, New York, NY, USA

## Abstract

**Background:**

The aim of this study was to quantify changes in body composition during ovarian cancer treatment and relate these changes to rates of complete gross resection (CGR).

**Methods:**

One hundred two patients with stage III or IV ovarian cancer who underwent neoadjuvant chemotherapy (NACT) followed by interval debulking surgery were a part of a prospectively collected database that included computed tomography scans at three time points—diagnosis, following NACT, and following debulking surgery. Skeletal muscle, visceral adipose, and subcutaneous adipose tissue volumes were obtained from a 30-mm volumetric slab beginning at the third lumbar vertebrae.

**Results:**

Following NACT, skeletal muscle volume was significantly reduced (352.5 to 335.0 cm^3^, *P* < 0.001), whereas adiposity was unchanged. Body mass index (BMI) and skeletal muscle volume were significantly lower in patients who achieved CGR (*P* < 0.05). When these patients were stratified by BMI, the significant association of skeletal muscle to CGR was limited to patients with a BMI < 25 kg/m^2^ (*P* = 0.007).

**Conclusion:**

Skeletal muscle volume was significantly reduced in patients undergoing NACT for ovarian cancer. Non-overweight patients were more likely to achieve CGR if they had lower skeletal muscle volume. Use of volumetric-based measurement for ascertaining body composition should be explored further.

## Introduction

Ovarian cancer is the leading cause of death in women with gynaecological cancer.^1^ The standard approach to treatment is primary debulking surgery followed by platinum-based and taxane chemotherapy in women who are eligible for surgery. neoadjuvant chemotherapy (NACT) followed by interval debulking surgery is an alternative to primary debulking surgery in patients who are not eligible for upfront surgery or in whom suboptimal debulking is expected due to the extent of disease. Two randomized trials reported similar survival rates among patients treated with NACT vs. primary debulking surgery followed by chemotherapy; however, survival in both arms of these studies was poorer than expected.^2,3^ Additional trials are ongoing.

Obesity is thought of as a trigger for the development ovarian cancer. For example, multiple cohort studies have demonstrated an association between ovarian cancer mortality and obesity, as measured by body mass index (BMI).^4,5^ One study found that the relative risk of death increased 7% for each 5% increase in BMI.^6^ This increased risk likely stems from physiologic changes resulting from excess adiposity, a clinical feature that typically correlates with BMI.^7–10^ BMI, however, may not accurately reflect whole body adiposity in patients with ovarian cancer because of the changes that occur in total body water, such as ascites or oedema. Therefore, it is not surprising that some groups have found no change in survival when examining BMI at the time of diagnosis.^11–13^ A more accurate measurement of adiposity can be obtained using computed tomography (CT) from standard of care imaging.^14–16^ This technique has shown that weight gain following debulking surgery is primarily body fat and that loss of skeletal muscle, as measured by sequential CT scans, is associated with decreased survival in ovarian cancer patients.^17,18^

The aim of the current study was to investigate changes in body composition, including changes in skeletal muscle, subcutaneous adipose tissue (SAT), and visceral adipose tissue (VAT) volumes, in ovarian cancer patients receiving NACT. Achieving surgical complete gross resection (CGR) is regarded as the most important prognostic factor in patients with advanced ovarian cancer^3,19,20^; therefore, we attempted to identify clinical and body composition parameters that are enriched in patients achieving CGR. Moreover, loss of skeletal muscle has been shown to be an independent prognostic factor in other cancers.^21^

## Methods

### Patients and setting

This is a retrospective analysis of prospectively collected clinical data and imaging parameters of a cohort of patients with a diagnosis of epithelial ovarian cancer treated with NACT and interval debulking surgery at Memorial Sloan Kettering Cancer Center from 1 January 2008 through 1 May 2013.^22^ CGR was defined as no grossly visible disease at the completion of surgery.

### Computed tomography image analysis

Quantities of skeletal muscle, VAT, and SAT were calculated from CT images using iNtuition software (v4.4, TeraRecon, Foster City, CA) by a single reader (J. V.) at three time points over the course of therapy—at diagnosis, following NACT, and following debulking surgery. The accuracy of CT tracing was verified by retracing a sample of the cohort at the end of the study. The average difference for all the measurements combined was 1%.

**Figure 1:**
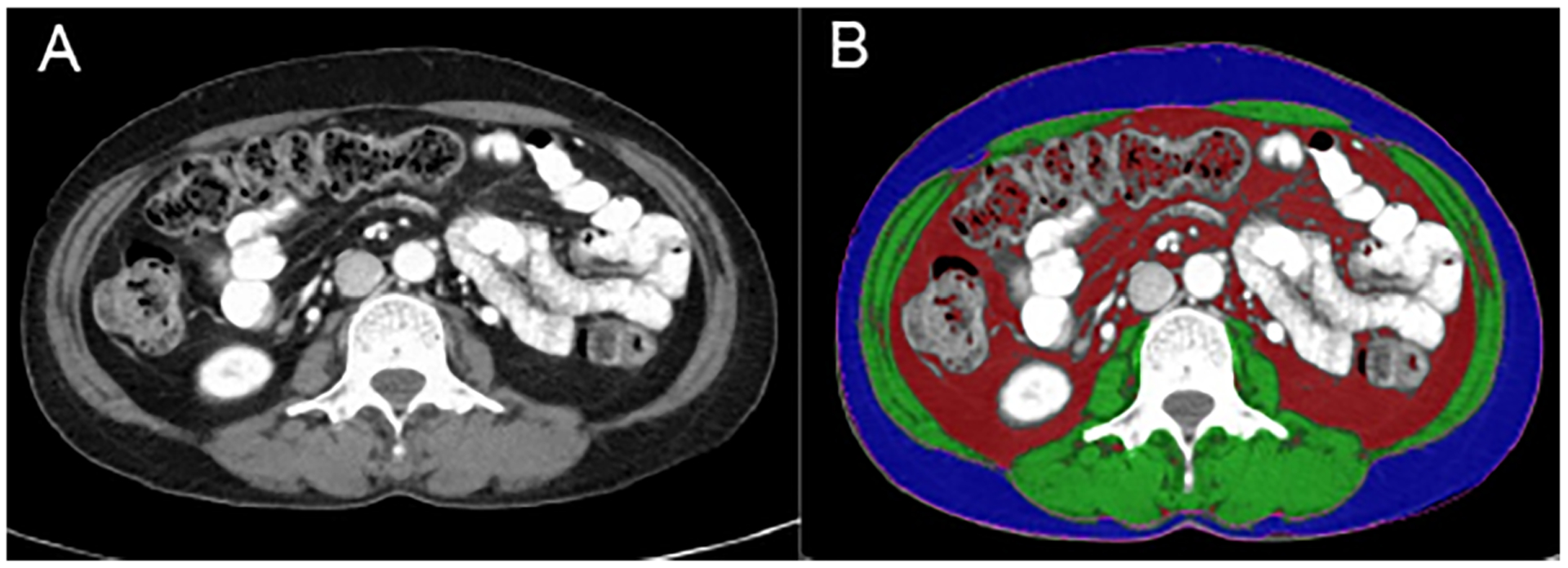
Semi-automated body composition analysis. (A) Example of an axial computed tomography slice at the level of L3. (B) Segmentation of body composition using iNtuition software. Green = skeletal muscle, blue = subcutaneous adipose tissue, red = visceral adipose tissue.

For each imaging data set, a 30-mm volumetric slab was analysed for the presence of skeletal muscle and adipose using a semi-automated technique. First, Hounsfield unit (HU) thresholds were set to −150 to −50 HU to identify adipose tissue and −29 to 150 HU to identify skeletal muscle, as previously described.^23^ A colour-coded map of voxels with the specified HU values was generated for six 5-mm thick CT levels. The non-muscular soft tissues (abdominopelvic viscera, large blood vessels, spinal cord, and portions of the bone marrow) were manually excluded by drawing a region of interest around the identified tissue region. The percentage of low-density muscle (HU < 0) was also recorded from each volumetric slab. SAT and VAT volumes were segmented using a similar semi-automated approach with iNutrition. An example of the semi-automated contouring is shown in Figure 1.

### Statistical measures

Patient demographic and clinical characteristics were summarized using descriptive statistics. Body composition metrics (skeletal muscle, SAT, and VAT volumes) were summarized at each time point, and per-patient percentage differences were calculated. Comparisons of each metric from the prior time point (diagnosis vs. following NACT and following NACT vs. following debulking surgery) were conducted using the nonparametric Wilcoxon signed rank test.

Patients were classified by the achievement of CGR vs. any residual disease during debulking surgery. Comparisons between these groups and clinical factors were examined using the Wilcoxon rank sum test for continuous variables and the Fisher exact test for categorical variables. Subset analyses based on the BMI categories [non-overweight (BMI < 25 kg/m^2^), overweight [BMI = 25–29 kg/m^2^], and obese (BMI ≥ 30 kg/m^2^)] were also conducted. Within each BMI category, skeletal muscle volume was compared with surgical residual groups using the Wilcoxon rank sum test. Logistic regression was used to further examine the relationship between BMI and skeletal muscle with surgical outcome (CGR). Area under the receiver operating characteristic curve (AUC) was used to measure the predictive ability of either BMI or skeletal muscle on surgical outcome.

All significance tests were two sided, with a 5% level of significance. Analyses were performed with SAS 9.4 (SAS Institute Inc).

### Results

One hundred fifty-four patients were evaluated over the study period; 52 were excluded for the following reasons: missing CT or clinical data, poor image quality (e.g. hardware scatter, motion, or windmill artefact), and atypical imaging parameters (unequal slice thickness or spacing), which were important for calculating volumetric data over multiple slices. The remaining 102 patients were included in the study, and their characteristics are described in Table 1. There was no difference between included and excluded subjects (Supporting Information, Table S1). The median age of the included subjects was 64 years (range, 38–90 years), and the median BMI was 25.7 kg/m^2^ (range, 17.4–49.8 kg/m^2^). The cohort was evenly split (50%) between stage IIIC and stage IV disease. Median preoperative CA-125 was 968 units/mL (range, 6–16,923 units/mL). Sixty patients (59%) achieved CGR following debulking surgery. The median day between completion of neoadjuvant therapy and interval debulking surgery was 29 days (Supporting Information, Table S2).

The median volumes of skeletal muscle, SAT, and VAT over the course of therapy (diagnosis, following NACT, and following debulking surgery) are shown in Table 2. Skeletal muscle volume was significantly reduced from diagnosis to following NACT (*P* < 0.001), whereas VAT and SAT volumes were unchanged. Following debulking surgery, which includes an omentectomy, VAT volume was significantly reduced (*P* < 0.001), whereas skeletal muscle and SAT volumes remained unchanged in comparison with the NACT scan.

To identify clinical parameters that are associated with CGR, patients were stratified based on CGR status (Table 3). Patients achieving CGR had significantly lower BMI (*P* < 0.05) and skeletal muscle volume at diagnosis (*P* < 0.05) and following NACT (*P* < 0.05). There was a trend for patients achieving CGR to have lower amounts of VAT (*P* = 0.058) as well. The association between skeletal muscle volume and CGR among different BMI subsets was also examined (Table 4). The significant association of skeletal muscle volume to CGR was found to be limited to patients with a BMI < 25 kg/m^2^ (*P* = 0.007). In a univariate logistic regression analysis, BMI and the skeletal muscle volume following NACT were inversely correlated with CGR [OR for BMI: 0.926 (95% CI: 0.861, 0.996), and skeletal muscle: 0.990 (95% CI: 0.982, 0.998)]. To determine if skeletal muscle is a better predictor of CGR outcome, we calculated the AUC. The AUC for both BMI [AUC 0.617 (95% CI: 0.508–0.726)] and skeletal muscle volume [(AUC 0.646 (95% CI: 0.537–0.755)] were similar. A bivariate logistic regression model was built to further investigate the relationship between BMI and CGR outcome after controlling for skeletal muscle. Based on the model, neither BMI (*P* = 0.34) nor skeletal muscle (*P* = 0.112) showed any significance.

## Discussion

This is the first study to use radiologic measurements to identify trends in volumetric skeletal muscle and adipose tissue over the course of ovarian cancer treatment. We found that patients undergoing NACT for advanced ovarian cancer lost skeletal muscle volume; however, adiposity remained unchanged. This is in keeping with other published reports of patients with ovarian cancer undergoing chemotherapy and support the concerning deleterious effect of cytotoxic agents on skeletal muscle.^17,18,24^ This effect may be due to the production of endogenous glucocorticoids, which elicits significant muscle atrophy in mice treated with cytotoxic chemotherapy.^25^

We hypothesized that patients with ovarian cancer and higher VAT would have lower rates of CGR due to the greater area available in which disease can spread and elude detection during surgery. Previous studies using BMI as a surrogate for excess adiposity (including VAT) have shown that obese patients experience increased surgical blood loss and longer operating room time; however, no clear association with CGR has been detected.^26,27^ In the current study, we directly measured the amount of VAT and SAT in patients achieving CGR to those with any residual disease. Although we did detect a trend towards lower volumetric VAT in patients achieving CGR, this finding did not reach statistical significance. The lack of significance was influenced by the high variability in the distribution of VAT among subjects. Given the overlap in CT density between adipose tissue and malignant ascites, the identification of true VAT using imaging parameters may not be accurate in this setting.

Interestingly, we found that BMI and volumetric skeletal muscle were significantly lower in patients achieving CGR. A further exploration of these findings revealed that the association of CGR with skeletal muscle volume was limited to non-overweight patients (BMI <25 kg/m^2^). One explanation for this finding is that in non-overweight patients, additional skeletal muscle makes disease detection and removal more difficult. With this knowledge, surgeons could better stratify patients’ risk and better strategize their operative plans to achieve CGR.

An important difference in our methodology from other body composition studies is the use of volumetric measurements instead of single axial slices. Volumetric measurement appears to be more accurate in assessing body composition. Studies estimating total volume of both visceral adipose and skeletal muscle found that more single axial slices on magnetic resonance imaging were needed to reach the same power of volumetric measurements.^16,28^ Although many body composition studies utilize single axial slices, volumetric measurements have been used in both non-small cell lung cancer and melanoma.^29,30^

Our study is limited by its retrospective nature, which can lead to selection bias. An additional limitation is that we only examined patients undergoing NACT and interval debulking surgery, and these patients tend to have more disease or comorbidities that prevent them from undergoing primary debulking surgery. Furthermore, patients with CT images containing artefacts or incorrect image spacing were excluded. It is possible, albeit unlikely, that patients with images with these technical difficulties comprise a unique population that is underrepresented in our cohort. Despite these limitations, this study was able to reliably measure changes in skeletal muscle and adipose tissue volumes to uncover novel associations in patients achieving CGR.

## Supplementary Material

Supporting Information Table S1. Patient Characteristics of Excluded Subjects (*N* = 52)
Table S2. The days between completion of neoadjuvant therapy and Interval Debulking Surgery

## Figures and Tables

**Table 1:** Patient characteristics (*N* = 102)

		Median	Range	No. of Patients	%
Age, years		64	38–90		
BMI, kg/m^2^		25.7	17.4–49.8		
Days between scans		89	39–196		
CA-125, units/mL		968	6–16,923		
Stage (FIGO 1988)	IIIC			51	50.0
	IV			48	47.1
	IVB			3	2.9
Medical comorbidities					
Hypertension				40	39.2
Pulmonary disease				18	17.6
Hypothyroid				13	12.7
Diabetes				12	11.8
Coronary artery disease				5	4.9
Complete Gross Resection				60	59.0

BMI, body mass index; FIGO, International Federation of Gynecology and Obstetrics.

**Table 2:** Changes in body composition during neoadjuvant and adjuvant therapy.

Tissue volume median (range)	Diagnosis	Following NACT	% change	Following surgery	% change
Muscle (cm^3^)	352.5 (197–497)	335.0 (233–473)*	−**2.5** (−23.7–56.3)	341.0 (222–552)	1.4 (−19.9–87.1)
VAT (cm^3^)	231.0 (13.4–982)	207.0 (11.1–933)	−1.3 (−75–189.2)	175.0 (24–878)**	−**10.1** (−60.4–184.7)
SAT (cm^3^)	542.5 (45.8–1,564)	583.0 (103–1,657)	−0.6 (−49–262.4)	576.5 (18.6–1,662)	0.7 (−94.7–250.6)

NACT, neoadjuvant chemotherapy; VAT, visceral adipose tissue; SAT, subcutaneous adipose tissue.

* *P* < 0.001 between diagnosis and the scan following NACT.

** P < 0.001 between NACT scan and the scan following debulking surgery using the Wilcoxon rank sum test.

**Table 3:** Association of complete gross resection with clinical factors and skeletal muscle volumes

	CGR		Any residual	*P* ^a^
Total patients	60		42	
Age at diagnosis, years				
Median (Mean)	64 (61.5)		65 (63.9)	0.266
Range	38–86		40–90	
BMI, kg/m^2^				
Median (Mean)	25.1 (25.8)		27.1 (28.3)	0.046
Range	17.4–40		19.4–49.8	
Stage				
IIIC	28 (46.7%)		23 (54.8%)	0.546
IV/IVB	32 (53.3%)		19 (45.2%)	
Preop CA125 (1 missing), units/mL				
Median (mean)	822 (1908.7)		1,150 (1738.8)	0.863
Range	6–16,923		34–10,500	
Comorbidities:				
Hypertension	24 (40%)		16 (38.1%)	1
Pulmonary disease	9 (15%)		9 (21.4%)	0.438
Hypothyroid	7 (11.7%)		6 (14.3%)	0.767
Diabetes	7 (11.7%)		5 (11.9%)	1
Coronary artery disease	5 (8.3%)		0 (0%)	-
Skeletal muscle volume, cm^3^				
Diagnosis				
Median (mean)	342 (343.6)		366 (365.9)	0.023
Range	238–497		197–497	
Following NACT				
Median (mean)	325 (330.1)		353.5 (356.4)	0.012
Range	241–461		233–473	
VAT volume, cm^3^				
Diagnosis				
Median (mean)	215 (220.1)		289 (290.7)	0.058
Range	24–575		13.4–982	
Following NACT				
Median (mean)	195.5 (216.6)		269.5 (285.3)	0.078
Range	34–721		11.1–933	
SAT volume, cm^3^				
Diagnosis				
Median (mean)	550 (608.1)	530 (630.8)		0.760
Range	45.8–1,478	96.5–1,564		
Following NACT				
Median (mean)	583 (608)		587 (631.5)	0.801
Range	107–1,657		103–1,433	

BMI, body mass index; CGR, complete gross resection; SAT, subcutaneous adipose tissue; VAT, visceral adipose tissue.

a *P* values were obtained using the Wilcoxon rank sum test for continuous variables and Fisher exact test for categorical variables.

**Table 4:** Relationship between muscle volume and complete gross resection by body mass index

BMI < 25 kg/m^2^ (*n* = 44)			
Following NACT	CGR	Any residual	*P* ^a^
Muscle volume, cm^3^			
Median (mean)	309 (306.7)	336 (342.7)	0.007
Range	241–377	298–473	
BMI: 25–30 kg/m^2^ (*n* = 31)			
Following NACT			
Muscle volume, cm^3^			
Median (mean)	327 (330.9)	321 (337.1)	1
Range	266–420	233–471	
BMI ≥ 30 kg/m^2^ (*n* = 27)			
Following NACT			
Muscle volume, cm^3^			
Median (mean)	371 (377.5)	397 (392.9)	0.396
Range	291–461	325–448	

BMI, body mass index; CGR, complete gross resection; NACT, neoadjuvant chemotherapy.

a *P* values obtained using the Wilcoxon rank sum test.
