# Supplementary material for: The effects of neoadjuvant chemotherapy and interval debulking surgery on body composition in patients with ovarian cancer
Source: JCSM Clin Rep. Author manuscript; Available in PMC 2021 Sep 3. (PMC8415724)
Supplement: Supporting Information — Table S1. Patient Characteristics of Excluded Subjects (N = 52) Table S2. The days between completion of neoadjuvant therapy and Interval Debulking Surgery [file NIHMS1729756-supplement-Supporting_Information.docx]

| **Supplemental Table 1. Patient Characteristics of Excluded Subjects (N=52)** | | | |  |
| --- | --- | --- | --- | --- |
|  | **Median** | **Range** | **Count** | **%** |
| Age, years | 68 | 34-89 |  |  |
| BMI | 25.1 | 19.1-42.2 |  |  |
| Days between scans | 122 | 0-221 |  |  |
| CA-125(4 missing) | 522 | 13-32100 |  |  |
| Stage |  |  |  |  |
| IIC |  |  | 1 | 1.9 |
| IIIC |  |  | 29 | 55.8 |
| IV |  |  | 22 | 42.3 |
| *Medical comorbidities* | |  |  |  |
| Hypertension (HTN) |  |  | 18 | 34.6 |
| Pulmonary disease |  |  | 11 | 21.2 |
| Hypothyroid |  |  | 8 | 15.4 |
| Diabetes (DM) |  |  | 3 | 5.8 |
| Coronary artery disease (CAD) | | | 2 | 3.8 |
|  | | |  |  |
| Complete Gross Resection (5 missing IDS residual) | | | 22 | 46.8 |

|  | | | | |
| --- | --- | --- | --- | --- |
| **Supplemental Table 2. The days between completion of neoadjuvant therapy and Interval Debulking Surgery** | | | |  |
|  | **All** | **CGR** | **Any Residual** |  |
| Median (Mean) | 29(30.8) | 29(31.4) | 30(30) |  |
| Range | 5-136 | 13-136 | 5-55 |  |
